# Effects of Chronic Bifidobacteria Administration in Adult Male Rats on Plasma Metabolites: A Preliminary Metabolomic Study

**DOI:** 10.3390/metabo12080762

**Published:** 2022-08-18

**Authors:** Francesca Biggio, Claudia Fattuoni, Maria Cristina Mostallino, Paolo Follesa

**Affiliations:** 1 Department of Life and Environmental Sciences, Section of Neuroscience and Anthropology, University of Cagliari, Monserrato, 09042 Cagliari, Italy; 2 Department of Chemical and Geological Sciences, University of Cagliari, Monserrato, 09042 Cagliari, Italy; 3 Institute of Neuroscience, National Research Council, Monserrato, 09042 Cagliari, Italy

**Keywords:** *microbiota*, *Bifidobacteria*, metabolomic, GC-MS

## Abstract

Probiotics are live microorganisms distributed in the gastrointestinal tract that confer health benefits to the host when administered in adequate amounts. Bifidobacteria have been widely tested as a therapeutic strategy in the prevention and treatment of a broad spectrum of gastrointestinal disorders as well as in the regulation of the “microbiota-gut-brain axis”. Metabolomic techniques can provide details in the study of molecular metabolic mechanisms involved in Bifidobacteria function through the analysis of metabolites that positively contribute to human health. This study was focused on the effects of the chronic assumption of a mixture of Bifidobacteria in adult male rats using a metabolomic approach. Plasma samples were collected at the end of treatment and analyzed with a gas chromatography-mass spectrometry (GC-MS) platform. Partial least square discriminant analysis (PLS-DA) was performed to compare the metabolic pattern in control and probiotic-treated rats. Our results show, in probiotic-treated animals, an increase in metabolites involved in the energetic cycle, such as glucose, erythrose, creatinine, taurine and glycolic acid, as well as 3-hydroxybutyric acid. This is an important metabolite of short-chain fatty acids (SCFA) with multitasking roles in energy circuit balance, and it has also been proposed to have a key role in the prevention and treatment of neurodegenerative diseases.

## 1. Introduction

Recently, an overwhelming interest by neuroscientists in probiotics related to human health has driven a growing number of studies to investigate their impact on the key mechanisms affecting the connection between gut microbiota and brain function, the so-called gut-brain axis.

The International Scientific Association defines probiotics as live microorganisms that, when administered in a balanced amount, lead to benefits for the host’s health [1].

In fact, there are many promising studies that elucidate the possible role of probiotics and the gut in controlling brain function using animal models compared to humans [2,3,4]. Gastrointestinal (gut) health has a crucial impact on human metabolism, producing and/or transforming circulating blood products, such as amino acids, fatty acids, bile acids, hormones and vitamins [5,6], mostly through its diverse community of microorganisms [7]. Furthermore, gut health seems, in part, to depend on short-chain fatty acids (SCFAs), the main metabolites produced in the colon via the bacterial fermentation of dietary fibers, which are considered to play a key role in neuro-immunoendocrine regulation through signals to the brain by activating afferent sensory neurons in the vagus nerve via neuroimmune and neuroendocrine pathways or by crossing the blood-brain barrier via systemic circulation [8]. However, what mechanisms through which SCFAs carry out their influence on brain physiology and behavior have not been yet been completely elucidated. The human gut microbiota includes a vast array of bacteria [9], and its alteration may cause an important relapse in gut microbiota health, leading both to “on-site” problems, such as irritable bowel syndrome (IBS), and central nervous system impairments. In agreement, several reports provide a wide and clear description of the role of the microbial community and its composition, which could strongly influence peripheral metabolic activity, as well as brain plasticity, increasing vulnerability to psychopathology when altered [10,11].

Recently, metabolomic studies have gained more interest in research since systemic biofluids, such as blood, urine and saliva, confer on each subject a unique and individual metabolic profile called a metabolic phenotype or “metabotype”. The metabotype, in homeostatic conditions, remains stable over time, allowing it to be a good reference to evaluate responses to various stimuli [12], as well as to better understand vulnerabilities to several diseases during development [13,14].

It is, therefore, clear that the metabotype provides multifactorial information, providing a specific characterization of genotype and metabolism that could also be affected by many factors, such as gut microbiota, diet, lifestyle and drug use [15].

Based on this evidence, many studies have focused on analyzing the differences in gut microbial community composition to elucidate how metabolic plasticity reflects the ability of a given community to adapt to environmental changes by regulating metabolic functioning.

Recently, we demonstrated that the chronic consumption of a mix of three different strains of nonpathogenic Bifidobacteria was able to increase the neuronal plasticity of the hippocampus, which was accompanied by a morphological modification of dendritic arborization and spine density, as well as an increase in neurotrophic factors and cognitive performance [16], further supporting the idea of a clear and controlled relationship between gut microbiota composition and brain function.

Although several studies have demonstrated that the daily intake of probiotics improves mucosal barrier function and positively alters the gut microbiota composition and intestinal metabolism [17,18], further investigations are needed.

Bifidobacteria are naturally present as dominant bacterial populations in the gastrointestinal tract of humans, representing up to 25% of the cultivable fecal bacteria in adults and 80% in infants. Commonly detected in the feces of healthy subjects, several Bifidobacterial species have been described so far, such as *B. longum*, *B. breve*, *B. infantis*, *B. bifidum*, *B. batenulatum*, *B. bseudocatenulatum* and *B. adolescentis* [19,20,21], which, during the human life span, differently modulate metabolic routes, immune activities and brain development and its function in the host [22]. Furthermore, it is now known how the growth and metabolic activity of probiotic bacteria, including Bifidobacteria, can be selectively stimulated by dietary compounds known as “prebiotics”, which are nondigestible food ingredients that benefit the host by selectively stimulating the growth or activity of one or a limited number of bacteria in the colon. Due to these properties and their capacity in gut-health promotion, specific Bifidobacteria strains are classified in specific categories and commercially sold as probiotic microorganisms for human health [23,24].

To further characterize this aspect, the principal aim of our research was to evaluate the effects of a chronic treatment (2 months) with a mix of Bifidobacteria (*B. longum*, *B. breve*, *B. infantis*) (B-Mix) on the metabolites and metabolic pathways of adult male rats.

Our results highlight the importance of using biological samples, such as blood, which can provide crucial information and are optimal sources for clinical diagnostic studies. In addition, rodents and humans exhibit a high grade of similarities with respect to the gastrointestinal tract [25] and are a good study model to better understand the role and the mechanisms in which probiotic interventions influence host health. Therefore, the analysis of the microbial community is important; it completes and enriches the evaluation of their function through metabolome examination.

## 2. Experimental Design

**Figure 1:**
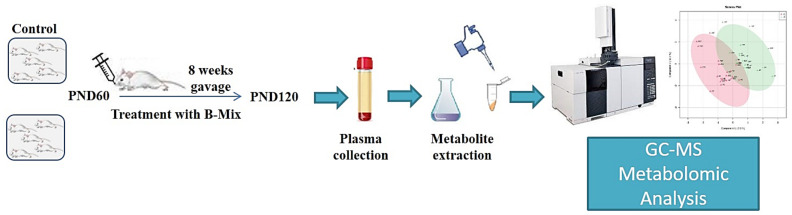
Experimental design scheme.

Rats at postnatal day 60 (PND 60) were divided into two experimental groups (Control and Probiotics) and started the treatment through gavage with a mix of Bifidobacteria for 8 weeks. At the end of treatment (PND 120), plasma was collected, and then metabolites were extracted and processed. Metabolites analyses were performed using an Agilent 5977B GC/MS (Figure 1).

## 3. Material and Methods

### 3.1. Animals

The study was performed using male Sprague-Dawley rats (200 g) (Charles River, Calco, Italy). All animals were housed in groups of four–five per cage (size: 60 cm × 38 cm × 18 cm) under an artificial 12 h light-dark cycle (8:00 a.m.–8:00 p.m.) at a controlled temperature (23 ± 2 °C) and humidity (65%). Food and water were available ad libitum. Animal care and handling throughout the experimental procedures were in accordance with the guidelines for the care and use of experimental animals of the European Communities Council (2010/63/UE L 276 20 October 2010) and with Italian law (DL: 4 March 2014, N° 26). The experimental protocols were also approved by the Animal Ethics Committee of the University of Cagliari, Italy. Furthermore, every effort was made to minimize suffering and reduce the number of animals used.

### 3.2. Bifidobacteria Treatment

Adult male rats were treated, at postnatal day 60 (PND 60), with gavage once a day (from 12:00 to 13:00) for 8 weeks with a mix of Bifidobacteria. Every day, animals were subject to a 4 h fasting before and 1 h after the treatment to obtain optimal gastric absorption. During the rest of the time, the rats were allowed to access chow ad libitum. Three different strains of Bifidobacteria (VALEAS S.p.a., Milano, IT) were used to prepare a fresh daily mix, as reported in Table 1: B. breve, M-16 V, 1 × 109 CFU/kg; B. longum, BB536, 3 × 109 CFU/kg; B. infantis, M-63, 1 × 109 CFU/kg. The proportions were expressed in colony-forming units (CFUs).

The mix was prepared from lyophilized single-bacteria strains (stored at 4 °C) dissolved in tap water right before the treatment and administered at 5 × 109 CFU/2 mL/kg of body mass. Control animals were treated by gavage once a day with the solvent solution of tap water alone.

### 3.3. Plasma Collection

Animals were sacrificed between 10:00 a.m. and 12:00 p.m. with a guillotine; blood was rapidly collected in K3-EDTA tubes and centrifuged at 1000× *g* for 10 min at 4 °C. Plasma fraction was transferred into new tubes and stored at −80 °C until GC-MS analysis.

### 3.4. Sample Preparation and GC-MS Analysis

Plasma samples were analyzed as previously reported for human samples [26] with slight modifications. In brief, 400 μL of plasma were treated with 1200 μL of cold methanol in 2 mL Eppendorf tubes, vortex mixed and centrifuged 10 min at 14000 rpm (16.9 × 1000 G). In total, 800 μL of the upper phase was transferred in glass vials (1.5 mL) and evaporated to dryness overnight in an Eppendorf vacuum centrifuge. Fifty μL of a 0.24 M (20 mg/mL) solution of methoxylamine hydrochloride in pyridine was added to each vial; samples were vortex mixed and heated in a dry-block heater at 80 °C for 15 min. Fifty μL of MSTFA (N-Methyl-N-trimethylsilyltrifluoroacetamide) was added and heated in a dry-block heater at 80 °C for 15 min. After cooling, the derivatized samples were diluted with hexane (100 μL). Analyses were performed on an Agilent 5977B GC/MS interfaced to the GC 7890B (Agilent Technologies, Palo Alto, CA, USA), equipped with a DB-5ms column (Agilent J&W Scientific, Folsom, CA, USA). Injector temperature was 230 °C, detector temperature was 280 °C, helium carrier gas flow rate was 1 mL/min. GC oven temperature program was the following: 90 °C initial temperature and 1 min hold time, increasing 10 °C/min to a final temperature of 270 °C, 7 min hold time. One μL of the derivatized sample was injected in split (1:4) mode. After a solvent delay of 4 min, mass spectra were acquired in full scan mode using 2.28 scans/s with a mass range of 50–700 Amu. Each acquired chromatogram was analyzed by means of the free software AMDIS (Automated Mass spectral Deconvolution and Identification System) (http://chemdata.nist.gov/mass-spc/amdis accessed on 28 March 2022), which identifies each chromatographic peak by comparing the relative mass spectra and the retention times with those stored in an in-house library comprising 255 metabolites. Other metabolites were identified using NIST14 (National Institute of Standards and Technology’s mass spectral database) and the Golm Metabolome Database (GMD) (http://gmd.mpimp-golm.mpg.de/ accessed on 28 March 2022). This strategy allowed for the detection of 101 compounds: following the identification levels defined by the Metabolomics Standards Initiative (MSI) [27], 97 were “confidently identified compounds” (level 1), 2 “putatively annotated compounds” (level 2), 1 “putatively annotated compound class” (level 3), 1 “unknown compounds”. The Excel data sheet obtained (101 metabolites × 40 samples) was submitted for statistical analysis.

### 3.5. Statistical Analysis

The data matrix was processed with the integrated web-based platform MetaboAnalyst 4.0 (http://www.metaboanalyst.ca/ accessed on 28 March 2022) [28]. Missing values were replaced with 1/5 of the minimum positive values in the original data; data were normalized by sum, log-transformed and Pareto scaled. Univariate analysis (*t*-test), partial least square discriminant analysis (PLS-DA) and orthogonal partial least square discriminant analysis (OPLS-DA) were obtained. PLS-DA models were tested with the leave-one-out cross-validation (LOOCV) method for the evaluation of statistical parameters (accuracy, correlation coefficient (R2), cross-validation coefficient (Q2)), which allowed us to determine the optimal number of components for the model description. Due to the tendency of PLS-DA to overfit data, all models were validated in order to understand whether the separation was statistically significant using the B/W permutation test [29].

## 4. Results

**Figure 2:**
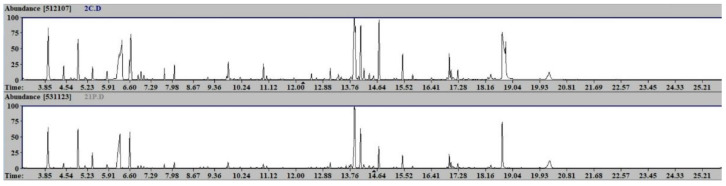
GC-MS chromatograms of the two experimental groups: upper chromatogram, control group; lower chromatogram, probiotic-treated group.

Forty plasma samples from probiotic-treated (20) and control rats (20) were analyzed. A representative chromatogram of the two experimental groups is shown in Figure 2. Statistical univariate analysis (*t*-test) did not reveal any significant metabolites.

**Figure 3:**
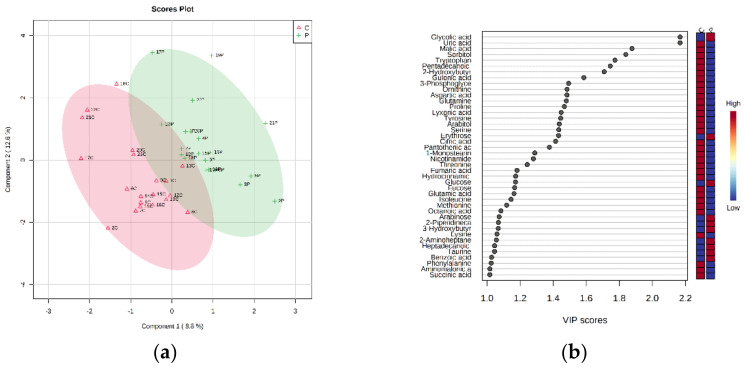
PLS-DA of plasma from control rats (C) vs. probiotic-treated rats (*P*). (**a**) Two-dimensional (2D) score plot and (**b**) the corresponding VIP score plot (threshold *p*-value >1.0).

The PLS-DA allowed us to build a model, whose score plot and VIP (variable importance in the projection) score plot (threshold *p*-value >1.0) are reported in Figure 3. The reported model was cross-validated (LOOCV), revealing a two-component model as the best attainable (accuracy = 0.725, R2 = 0.838, Q2 = 0.23452). Due to the tendency of PLS-DA to overfit data, the model was validated to understand whether the separation was statistically significant using the B/W permutation test, obtaining an empirical *p*-value of 0.13. As can be seen from the VIP score plot (Figure 3b), all resultant metabolites were more abundant in the control animals (C) except for glycolic acid, erythrose, glucose, arabinose, 2-piperidinecarboxylic acid, 3-hydroxybutyric acid, 2-aminoheptanedioic acid, heptadecanoic acid, taurine and benzoic acid. The metabolites were found to be more abundant in control rats, including 13 amino acids (tryptophan, ornithine (GC-MS product derived from arginine), aspartic acid, glutamine, proline, tyrosine, serine, threonine, glutamic acid, isoleucine, methionine, lysine, phenylalanine) and Krebs cycle intermediates such as malic, citric, fumaric and succinic acid. The PLS-DA VIP metabolites are reported in Table 2.

**Figure 4:**
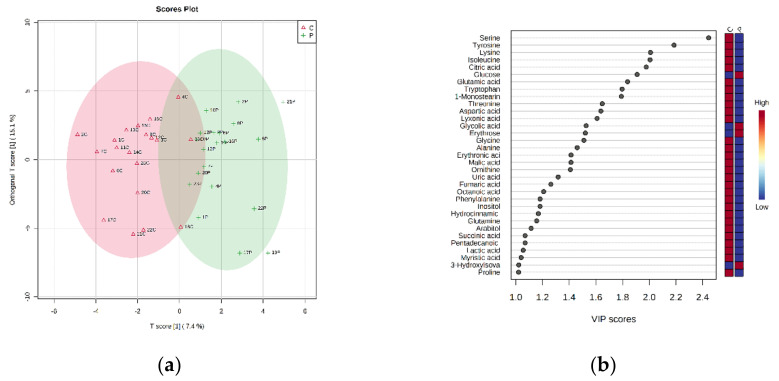
OPLS-DA of plasma from control rats (C) vs. probiotic-treated rats (P). (**a**) Two-dimensional (2D) score plot and (**b**) the corresponding VIP score plot (threshold *p*-value > 1.0).

An OPLS-DA allowed to build a model (R2X = 0.0737, R2Y = 0.507, Q2 = 0.19), whose score plot and VIP score plot (threshold *p*-value > 1.0) are reported in Figure 4.

The VIP score plot (Figure 4b) clearly shows all metabolites to be more abundant in the control group (C), except for glucose, glycolic acid, erythrose and 3-hydroxyisovaleric acid. These results confirm what was revealed by the PLS-DA.

## 5. Discussion

Great and important progress has been made in the last decade in terms of characterizing the bidirectional connection between the function of the central and enteric nervous systems, including the gastrointestinal tract. Through the use of different and elegant approaches, a series of preclinical studies have elucidated and described the crucial role of the gut microbiota in brain function through the well-known gut–brain axis [30,31]. In addition, there is strong evidence of the tight correlation between the peripheral dysregulation of gut microbiota and different brain disorders in humans [32], stimulating a growing interest in the involvement of metabolic compounds that may support gut composition and its activity through diet.

Here, with a metabolomic analysis, we studied the potential beneficial effect of the prolonged (2 months) consumption of three strains of Bifidobacteria (B-Mix) on metabolite balance in adult rats. Metabolomics is defined as the quantitative analysis of many metabolites with low molecular weights that are intermediate or final products of several metabolic pathways in living animals. Each metabolic profile detectable in a human biological fluid helps the scientific community to identify specific variations in metabolites involved in several disorders, which can be used to find a specific and efficacious treatment [33].

Our results provided the relative quantification of a total of 101 different metabolites, revealing a significant difference in B-Mix-treated animals when compared with their controls in 42 metabolites, mainly belonging to amino acid-, carbohydrate- and SCFA-related metabolism. Our data showed that the amount of several essential and nonessential amino acids—namely, tryptophan, arginine, aspartic acid, glutamine, proline, tyrosine, serine, threonine, glutamic acid, isoleucine, methionine, lysine and phenylalanine—is significantly reduced in B-Mix animals, while molecules such as glucose, erytrose, 3-hydroxybutyric, taurine and glycolic acid are increased.

Specific Bifidobacteria are involved in health-promoting functions [34]. It is now established that over 8% of the bifidobacterial-identified genes are predicted to be mainly involved in carbohydrate metabolism, showing an activity, for these compounds, greater than 30% compared to those used by other gut microorganisms [35,36,37,38].

Our results agree with the evidence that Bifidobacteria largely use carbohydrates as substrates to supply and optimize the body’s energy balance, using different amino acids that become more available and ready for Krebs or Urea cycles as well as for glucogenic and ketogenic processes. For instance, the Krebs cycle is not only responsible for most of the energy needs in complex organisms, but the molecules, produced in the cycle of chemical reactions, can be used as building blocks for a large number of important processes, including the synthesis of fatty acids, steroids, cholesterol, amino acids for building proteins and pyrimidines used in the synthesis of DNA. The ”fuel” for the Krebs cycle, which occurs in the mitochondria of eukaryotic cells, comes from lipids (fats) and carbohydrates, which both produce the molecules, such as acetyl coenzyme-A (acetyl-CoA). In B-Mix-treated animals, we found a high concentration of glucose, erythrose, arabinose and taurine, metabolites that come from amino acid catabolism, included in glucogenic and ketogenic circuits, suggesting that the increase in such strains of Bifidobacteria probably raise the rate of gluconeogenesis, rendering the energetic balance in these animals more efficient when compared with controls.

Recent studies describe a probiotic function predominately in the large intestine, and specific probiotic strains have their own proteolytic properties and have been associated with the increased production of digestive enzymes, improving, subsequently, host protein absorption and utilization [39]. In addition, probiotics offer various health benefits for stressed and active people [40], such as improving the integrity of the gut barrier function [41], and the administration of selected anti-inflammatory probiotic strains has been selected to improve a faster recovery from muscle-damaging exercise [42].

Another compound that we found significantly higher in animals treated with B-Mix was benzoic acid (BA). BA belongs to the aromatic carboxylic acid synthesized by the gut microbiota through the fermentation of dietary aromatic compounds. It is often used as a food preservative (E211) in the food and feed industry, such as for sodas and ready-made meals, and it is able to inhibit pathogenic microorganisms [43,44]. Some evidence suggests that the presence of BA is involved in gut health and improves functions, probably through its inhibiting action on diarrhea induced by *E. coli* infection [45,46].

As previously described, the microbial populations within gut microbiota coexist in a sensitive balance that can be affected by several perturbations, such as those imposed by antibiotic treatments and enteropathogen challenges or dietary compounds (style) such as nondigestible carbohydrates [47]. In vitro and in vivo studies have reported that by modulating the bifidobacterial population through probiotic or prebiotic supplements, it is possible to modify the overall composition and metabolism of gut microbiota [48,49]. For instance, several metabolic mechanisms are affected by supplementation with a *B. longum* strain, increasing the production of pymelate, butyrate and biotin metabolites in a human-gut-derived microbiota mouse model [49]. In the last decade, considerable effort has been invested in understanding gut microbiome status and functions using metabolic modeling, which generates testable hypotheses to elucidate the metabolism of individual species and interspecies metabolic interactions [50,51,52].

In our results, we found 3-hydroxybutyrate (3-HB), one of the most important short-chain fatty acids (SCFAs), significantly increased in B-Mix-treated animals compared with the respective control. 3-HB is a very important carboxylic acid intermediate metabolite of fatty acids found in animals, bacteria and plants. 3-HB is a product of the normal metabolism of fatty acid oxidation and can be used as an energy source in the absence of sufficient blood glucose. In fact, 3-HB has multiple important functions as a regulator in several circuits involving coenzyme A (CoA), free fatty acid receptor 3 (FFAR3), gamma-aminobutyric acid (GABA), histone deacetylases (HDAC), hydroxycarboxylic acid receptor 2 (HCAR2), nicotinamide adenine dinucleotide (NAD) and vesicular glutamate transporter (VGLUT) [53]. Although 3-HB is structurally similar to the neurotransmitter GABA (gamma-aminobutyric acid), no direct evidence of the effect of 3-HB on GABA receptors was found. However, the metabolism of 3-HB influences the production of GABA by increasing the synthesis of glutamate [54,55,56], thus also playing an important role in the balance of these opposite neurotransmitter systems. Several lines of evidence suggest that brain function and behavior are strongly influenced by microbial metabolites produced directly in the gut, such as 3-HB [57] and its metabolites, which are considered important epigenetic regulators that could influence long-lasting/stable host gene expression. In the brain, these metabolites could induce epigenetic changes in key genes responsible for the neuroprotection/regeneration mechanisms in psychiatric diseases. Particularly, butyrate is a known histone deacetylase (HDAC) inhibitor that blocks HDAC-mediated histone deacetylation. The hyperacetylation of histones enhances chromatin accessibility and activates gene expression in the neurons and glial cells of the central nervous system (CNS). This has been shown to facilitate long-term memory consolidation and neuroprotection/regeneration in different in vitro studies and animal models of learning, as well as in memory and neurodegenerative diseases [53,58].

Based on these considerations, HB has been proposed to play a key role in the prevention and treatment of neurodegenerative diseases such as Alzheimer’s, Parkinson’s and Huntington’s [59,60]. In animal model studies of neurodegenerative diseases, the hallmark role of 3-HB acid in reducing some characteristic symptoms has been well described, for example, preventing the accumulation of β-amyloid peptide, responsible of inflammation and oxidative stress in Alzheimer’s patients [61]; preventing the loss of dopaminergic neurons in the substantia nigra in Parkinson’s [62,63]; and improving the abnormalities in mitochondrial function and microglia overactivation in the brain in Huntington’s disorder [64,65].

The involvement of 3-HB in the etiology of depression is noteworthy. It is well known that the pathophysiology of depression is strongly associated with the inflammation of the nervous system. Brain areas, such as the prefrontal cortex, which is strongly involved in depression problems, seem to be a target of 3-HB, producing an antidepressant effect through anti-inflammatory mechanisms [66].

For this reason, butyrate is today used as an experimental treatment for neurological disorders such as depression, neurodegenerative diseases and cognitive impairments. This suggests that butyrate and other volatile SCFAs produced by gut microbes may be directly involved in the regulation of brain functions.

Studies with probiotic implementation containing Bifidobacteria have shown an extensive range of biological functions, especially in amino acid metabolism, carbohydrate metabolism, SCFA-related metabolism, the metabolism of cofactors and vitamins, secondary metabolites biosynthesis metabolism and genetic information. Acetate, butyrate and propionate are the most common SCFAs. SCFAs can inhibit the intestinal barrier lesion and prevent lactic acid from being transported into the bloodstream. Results have suggested a high concentration of SCFAs is present, further supporting the enhancement of carbohydrate digestion and absorption capacity. In fact, glycolysis/gluconeogenesis is considered the main carbohydrate metabolism that can generate SCFAs to repair the intestinal barrier and produce ATP to offer energy for growth [67].

Finally, we found a significant increase in heptadecanoic acids in animals after B-Mix treatment. The odd-chain fatty acids (OCFAs) pentadecanoic acid (15:0) and heptadecanoic acid (17:0) are the main odd-chain fatty acids in human plasma and are used as biomarkers for dairy-fat food intake assessment and disease risks. However, they can also be synthesized endogenously, for example, from gut-derived propionic acid (3:0). Beyond bacterial synthesis in the gut, endogenous propionic acid can originate from the degradation of amino acids such as methionine, valine, isoleucine and threonine. Their chains can be used to synthesize propionyl-coenzyme A (CoA). Propionyl-CoA comes from succinyl-CoA, and can replenish the citric acid cycle (CAC) with anaplerotic intermediates and, thus, improve mitochondrial energy metabolism. Mitochondrial function is impaired in several neurological disorders and may be reduced with increasing age.

A number of studies have established that a higher presence of OCFAs in plasma is associated with a lower risk of numerous diseases, such as metabolic syndrome [68]; decreasing fasting blood glucose levels [69,70]; improving insulin resistance [71]; and a lower incidence of type-2-diabetes mellitus [72,73,74]. In conclusion, the main focus of this study was to evaluate how the prolonged administration of probiotics affects specific blood metabolites. Indeed, we highlighted that a good balance and the proper functioning of the intestinal microbiome also depend on nutrition, revealing the fundamental role of traditional diets that provide starch, fiber and SCFAs such as butyrate.

Luckily, the gastrointestinal tracts of humans and rodents present a high grade of similarities, including the anatomy and the strain microbiota composition [25,75]. This aspect makes such animal models an optimal multitasking approach to better understand the role of microbiota in human health.

The human metabolic phenotype could reveal a very thorough and dynamic mirror of health, to help in comprehending the human organism mechanism and its function in adapting to processes [76,77].

## 6. Conclusions

Many reports have elucidated the role and tight relation between the gut and brain, suggesting that changes in gut microbiota could lead to modifications in several brain areas, including the modification of gene expressions involved in behavioral or psychiatric conditions such as mood disorders. 

Nevertheless, new studies will be needed for further investigation, which will help us to better understand how bacteria confer, with several mechanisms and metabolic methods, many of their positive effects on the host through the production of several metabolites. In the near future, a deeper understanding via metabolomic studies will help to further elucidate the biochemical mechanism by which probiotics function and pave the way toward more clinical and personalized applications.

## Figures and Tables

**Table 1:** Strain of bacteria tested and proportion of each strain in the mix used in the treatment.

Bifidobacterium Strain	CFU/kg/Day	Proportion
Bifidobacterium longum BB536	(3 × 109 CFU)	3/5 of total
Bifidobacterium breve M-16V	(1 × 109 CFU)	1/5 of total
Bifidobacterium infantis M-63	(1 × 109 CFU)	1/5 of total

**Table 2:** PLS-DA of the most important metabolites (VIP = variable importance in the projection; VIP score > 1), their chemical classification and their HMDB ID (https://hmdb.ca/ accessed on 28 March 2022).

Metabolite	Class ^b^	HMDB ID
Glycolic acid	HA	HMDB0000115
Uric acid	P	HMDB0000289
Malic acid	HA	HMDB0000156
Sorbitol	PO	HMDB0000247
Tryptophan	AA	HMDB0000929
Pentadecanoic acid ^a^	FA	HMDB0000826
2-Hydroxybutyric acid	HA	HMDB0000008
Gulonic acid	HA	HMDB0003290
3-Phosphoglyceric acid	HA	HMDB0000807
Ornithine	AA	HMDB0000214
Aspartic acid	AA	HMDB0000191
Glutamine	AA	HMDB0000641
Proline	AA	HMDB0000162
Lyxonic acid ^a^	HA	HMDB0060255
Tyrosine	AA	HMDB0000158
Arabitol	PO	HMDB0000568
Serine	AA	HMDB0000187
Erythrose	S	HMDB0002649
Citric acid	HA	HMDB0000094
Pantothenic acid ^a^	HA	HMDB0000210
1-Monostearin	MG	HMDB0011131
Nicotinamide	Am	HMDB0001406
Threonine	AA	HMDB0000167
Fumaric acid	A	HMDB0000134
Hydrocinnamic acid ^a^	A	HMDB0000764
Glucose	S	HMDB0000122
Fucose	S	HMDB0000174
Glutamic acid	AA	HMDB0000148
Isoleucine	AA	HMDB0000172
Methionine	AA	HMDB0000696
Octanoic acid	FA	HMDB0000482
Arabinose	S	HMDB0029942
2-Piperidinecarboxylic acid	A	HMDB0000070
3-Hydroxybutyric acid ^a^	HA	HMDB0000011
Lysine	AA	HMDB0000182
2-Aminoheptanedioic acid ^a^	AA	HMDB0034252
Heptadecanoic acid	FA	HMDB0002259
Taurine	AA	HMDB0000251
Benzoic acid	A	HMDB0001870
Phenylalanine	AA	HMDB0000159
Aminomalonic acid	AA	HMDB0001147
Succinic acid	A	HMDB0000254

^a^ Identification level 2 (all other metabolites were identified at level 1). ^b^ Chemical class: HA (hydroxy acid), P (purine), PO (polyol), AA (amino acid), FA (fatty acid), S (sugar), MG (monoglyceride), Am (amide), A (acid).

## Data Availability

The data presented in this study are available in the main article.
